# *Mycobacterium chelonae* empyema with bronchopleural fistula in an immunocompetent patient

**DOI:** 10.4103/1817-1737.56004

**Published:** 2009

**Authors:** Siraj Wali

**Affiliations:** *Department of Medicine, College of Medicine, King Abdulaziz University, Jeddah - 215 89, Saudi Arabia*

**Keywords:** Bronchopleural fistula, empyema, *Mycobacterium chelonae*, nontuberculous mycobacteria, rapidly growing mycobacteria

## Abstract

*Mycobacterium chelonae* is one of the rapidly growing mycobacteria that rarely cause lung disease. *M chelonae* more commonly causes skin and soft tissue infections primarily in immunosuppressed individuals. Thoracic empyema caused by rapidly growing mycobacteria and complicated with bronchopleural fistula is rarely reported, especially in immunocompetent patients. In this article we report the first immunocompetent Arabian patient presented with *M chelonae*-related empyema with bronchopleural fistula which mimics, clinically and radiologically, empyema caused by *Mycobacterium tuberculosis*.

Rapidly growing mycobacteria (RGM) are worldwide environmental organizms which are able to form colonies in less than one week.[1] Pulmonary disease related to RGM is predominantly due to mycobacterium abscessus and mycobacterium fortuitum.[2] *Mycobacterium chelonae* is a rare respiratory pathogen but more commonly causes skin and soft-tissue infections.[3]

Thoracic empyema caused by RGM and complicated with bronchopleural fistula is rarely reported. In this article, we report the first Arabian case with *M chelonae* empyema with bronchopleural fistula.

## Case Report

A 39-year old Saudi lady, with history of pulmonary tuberculosis more than 10 years ago, admitted to King Abdulaziz University Hospital in July 2008. She had a six-month history of weight loss, night sweat, dyspnea class II, productive cough with scanty yellow sputum and occasional mild hemoptysis. The patient was thin, with kypho-scoliosis but comfortable and vital signs were all within normal. Chest examination revealed bronchial breath with crackles over right base.

**Figure 1:**
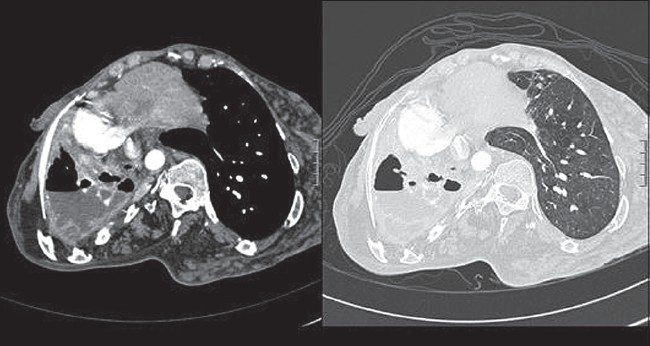
Computed tomography of the chest revealed marked collapse of the right lung, ipsilateral shift of mediastinum with bronchiectatic changes within the collapsed lung. It also showed thick and calcified rind, loculated pleural effusion with air-fluid level and right sided rib thickening. The left lung parenchyma was normal

Complete blood count showed normochromic normocytic anemia of 10.7g /dl otherwise normal. Both liver and renal function tests were normal. Erythrocyte sedimentation rate was 100. Human immunodeficiency virus serology was negative. Chest radiography showed severe scoliosis with lucency and an air-fluid level within dense lenticular pleural calcifications on the right hemithorax. Computed Tomography (CT) of the chest revealed marked collapse of the right lung with ipsilateral shift of the mediastinum. There were right sided thick and calcified rind, loculated pleural effusion with air-fluid level and rib thickening[Figure 1]. Normal left lung parenchyma.

Mycobacterium tuberculosis (MTB) empyema with bronchopleural fistula was suspected. Accordingly, four antituberculous medications; isoniazid, rifampicin, pyrazinamide and ethambutol were started empirically.

Three sputum specimens and a thick-yellow pleural fluid sample were collected. Specimens were decontaminated and concentrated. Direct smear from the deposit of each specimen was performed using Ziehl-Neelsen (ZN) stain. All samples were positive for acid fast bacilli (AFB). The remaining deposit was cultured on Lowenstein Jensen's (LJ) medium and BacT/Alert tuberculous liquid culture bottles (Organon Teknika; Durham N.C.). LJ cultures were incubated for eight weeks and BacT/Alert tuberculous liquid cultures were incubated for 14 days. Smears from positive culture were also performed using ZN stain for confirmation of AFB. Molecular assays were performed on all isolates for MTB deoxyribonucleic acid (DNA) using Amplicor Polymerase Chain Reaction (PCR) MTB assay (Roche Diagnostic Systems, Branchburg, NJ) according to manufacturer's instructions. Mycobacterium species with PCR MTB DNA negative were isolated from all samples within seven days indicating RGM. Speciation of mycobacterial isolates was carried out by the Multi-Gen Detection System (Genotype Mycobacteria) by Symbiosis (Via San Carlo, 10, 14023 Cocconata, Asti, Italy) according to manufacturer's instructions. Briefly, DNA of the mycobacteria was amplified with biotin-labeled primers. After denaturation, the DNA was hybridized with probes for the different mycobacteria species. A reading card was used for interpretation of the various bands obtained. Isolates from both sputum and pleural fluid were proven to be *M chelonae*. Susceptibility test showed that *M chelonae* was sensitive to Clarithromycin, Ciprofloxacin and Amikacin

The final diagnosis is *M chelonae* empyema with bronchopleural fistula. Clarithromycin, moxifloxacine and amikacin were started. Amikacin was continued for 12 weeks. All anti-TB medications were discontinued. Decortications was considered but declined by the patient who then lost follow up.

## Discussion

Rapidly growing mycobacteria include three clinically relevant species *M fortuitum*, *M chelonae*, and *M abscessus*. They are generally considered normal inhabitants of the environment and are found in water, soil, aerosol, wild and domestic animals, and fish.[4,5] RGM can cause lung disease in patients with and without underlying lung disease.[2] RGM most commonly complicate lung disease due to previous mycobacterial disease, CF, malignancy, COPD, lipoid pneumonia, and conditions associated with chronic gastro esophageal reflux, or vomiting.[2,6–8] Griffen *et al.* reviewed the clinical features of pulmonary disease caused by RGM in 154 cases.[2] Specific underlying diseases were infrequent, but they included previously treated mycobacterial disease (18%), coexistent Mycobacterium avium complex (eight per cent), cystic fibrosis (six per cent), and gastro esophageal disorders with chronic vomiting (six per cent).[2] Pulmonary disease due to RGM is predominantly due to *M abscessus* (80 per cent of cases) and *M fortuitum* (15 per cent of cases).[2] In contrast, *M chelonae* tends to cause skin and soft tissue infection and does not affect the lung.[3]

Thoracic empyema complicated with bronchopleural fistula is rarely reported to be caused by RGM including *M chelonae*, especially in immunocompetent patients. Most of these rare cases were actually reported from Far East and were due to infection caused by *M fortuitum* and *M abscessus*.[9] However, Hsieh *et al.* recently reported a 53-year old otherwise healthy woman with a right thoracic empyema and bronchopleural fistula and to our knowledge this is the only patient reported in the English literature to be due to *M chelonae*.[9] Like our patient, the pathogens from both pus and sputum were identified as *M chelonae*.

Our patient had clinical and radiological presentation similar to that of MTB empyema with bronchopleural fistula. The later is a rare manifestation of pleural tuberculosis that may be present for years with paucity of symptoms due to marked pleural thickening which confines the AFB.[10] Bronchopleural fistula complicating MTB empyema may present acutely with fever, dyspnea and copious sputum, or chronically with insidious onset of fatigue and constitutional symptoms.[10] In our present case, it is important to consider initially MTB as the underlying pathogen till proven otherwise since MTB is infectious and epidemic in Saudi Arabia.

*M chelonae* organizms are characterized by a high degree of *in vitro* resistance to antituberculous drugs.[3] Consequently susceptibility testing should be performed with antibacterial drugs including amikacin, doxycycline, imipenem, fluoroquinolones, a sulfonamide, cefoxitin, and clarithromycin rather than antituberculous medications. Unfortunately, there are no published controlled clinical trials of treatment comparing one form of treatment with another or with no drug treatment at all. Hence, treatment recommendations are based on case series and *in vitro* susceptibility in addition to experts' opinion.[11] In general, clarithromycin has become the mainstay of oral therapy for RGM. However, it is crucial to treat these infections with combination antimicrobial therapy based on the susceptibility pattern, to avoid resistance to macrolides following monotherapy. Although the optimal duration of therapy is not well defined, treatment for a minimum of 12 months of sputum-negative patients should be considered.[2,12] Surgery can be curative and may be contemplated in selected cases if the lung infection is localized and the patient performance status allows surgical approach.[12,13]
